# Genome-Wide Expression and Physiological Profiling of Pearl Millet Genotype Reveal the Biological Pathways and Various Gene Clusters Underlying Salt Resistance

**DOI:** 10.3389/fpls.2022.849618

**Published:** 2022-03-28

**Authors:** Samrah Afzal Awan, Imran Khan, Rezwan Tariq, Muhammad Rizwan, Xiaoshan Wang, Xinquan Zhang, Linkai Huang

**Affiliations:** ^1^College of Grassland Science and Technology, Sichuan Agricultural University, Chengdu, China; ^2^Department of Plant Protection, Akdeniz University, Antalya, Turkey; ^3^Department of Environmental Sciences and Engineering, Government College University Faisalabad, Faisalabad, Pakistan

**Keywords:** pearl millet, salinity, transcriptome profiling, transcripts, mechanism

## Abstract

Pearl millet (*Pennisetum glaucum* L.) is a vital staple food and an important cereal crop used as food, feed, and forage. It can withstand heat and drought due to the presence of some unique genes; however, the mechanism of salt stress has been missing in pearl millet until now. Therefore, we conducted a comparative transcriptome profiling to reveal the differentially expressed transcripts (DETs) associated with salt stress in pearl millet at different time points, such as 1, 3, and 7 h, of salt treatment. The physiological results suggested that salt stress significantly increased proline, malondialdehyde (MDA) content, and hydrogen peroxide (H_2_O_2_) in pearl millet at 1, 3, and 7 h of salt treatment. In addition, pearl millet plants regulated the activities of superoxide dismutase, catalase, and peroxidase to lessen the impact of salinity. The transcriptomic results depicted that salt stress upregulated and downregulated the expression of various transcripts involved in different metabolic functions. At 1 and 7 h of salt treatment, most of the transcripts were highly upregulated as compared to the 3 h treatment. Moreover, among commonly enriched Kyoto Encyclopedia of Genes and Genomes (KEGG) pathways, the mitogen-activated protein kinase (MAPK) signaling pathway and peroxisome pathway were significantly enriched. The DETs related to hormone signaling (auxins, ethylene, gibberellin, and abscisic acid), kinases, protein modifications, and degradation were also identified, depicting the possible role of hormones and kinases to enhance plant tolerance against salt stress. Furthermore, the transcription factors, such as ethylene-responsive element binding factors (ERF), basic helix-loop-helix (bHLH), HMG box-containing protein (HBP), MADS, myeloblastosis (MYB), and WRKY, were predicted to significantly regulate different transcripts involved in salt stress responses at three different time points. Overall, this study will provide new insights to better understand the salt stress regulation mechanisms in pearl millet to improve its resistance against salinity and to identify new transcripts that control these mechanisms in other cereals.

## Introduction

Agriculture faces tremendous pressure in human history due to immense climatic changes worldwide. Abiotic stresses (salinity, drought, heat, cold, etc.) contribute a wide range of harsh impacts on various crops in arid and semi-arid regions (Varshney et al., 2017). Salinization is an important issue in these areas caused by the regular use of irrigated water containing salts in the form of sodium chloride (NaCl) or sodium bicarbonate (NaHCO_3_). At early stages, salinity stress causes more sodium (Na^+^) and chloride (Cl^–^) ions uptake in different plant parts, which later severely impairs the plant growth at the reproductive stage and ultimately reduces productivity (Munns and Tester, 2008; Kamran et al., 2020). Furthermore, salinity leads to increased malondialdehyde (MDA) and reactive oxygen species (ROS) in plants that can severely impact the plants. However, salt-tolerant species have been reported to increase the activities of antioxidant enzymes, such as superoxide dismutase (SOD), catalase (CAT), peroxidase (POD), and ascorbate peroxidase (APX), to tolerate and survive under salinity (Khan et al., 2021b; Kumar et al., 2021).

Transcription factors (TFs), as master regulators, activate or repress the genes *via* binding to stress-related *cis*-elements in the promoter regions of downstream targeted genes (Gujjar et al., 2014; Liu et al., 2014). The insights behind the TF regulatory mechanisms facilitate the series of signaling networks in plants against stress responses (Shao et al., 2015). In the recent era, the networks of TFs related to responses against different environmental stresses are unraveling, and several TF-encoding genes involved in the responses against various types of abiotic stresses have been identified and reported (Jiang et al., 2018). Among various regulatory genes, stress-related TFs, i.e., Basic leucine zipper (bZIP), Basic helix-loop-helix (bHLH), APETALA2/ethylene-responsive element binding factors (AP2/ERF), NAC (NAM, ATAF, and CUC), WRKYs, etc., seemed to be repressed or induced by different abiotic stresses. However, overexpression of a few TFs improves the tolerance in transgenic plants. For instance, the *ERF76*, which is remarkably over-expressed in leaves, roots, and stems, enhanced the salt stress tolerance in *Populus simonii* × *Populus nigra* by regulating the 375 differentially expressed genes (DEGs), including 107 downregulated and 268 upregulated genes (Yao et al., 2016). Moreover, TFs have been suggested to regulate different genes that participate in different biological pathways. For example, *MYB9* modulates the flavonoid biosynthesis pathway in Epimedium (Huang et al., 2017) that may interact with the rest of the genes to participate in the respective biological pathway. Moreover, WRKY genes in wheat and AP/bZIP in maize have been identified on exposure to different types of stresses such as salinity, drought, cold, abscisic acid (ABA), gibberellic acid (GA), and hydrogen peroxide (H_2_O_2_), and have been involved in improving the tolerance of transgenic plants against these stresses by regulating various physiological, biochemical, and gene expression patterns (Wang et al., 2015, 2017). Furthermore, it has been investigated that WRKY transcription factors are involved in sugar signaling, hormone signaling, root growth, trichome development, senescence, pathogen defense, and other abiotic stresses (Zhou et al., 2008; Luo et al., 2013; Wang et al., 2015). In addition, it has been stated that AP2/ERF genes are induced in response to salt stress in durum wheat (Faraji et al., 2020). Besides, the potassium channel genes such as AKTs and KATs also have important functions in plant responses to abiotic stresses such as salinity (Musavizadeh et al., 2021). However, there is little information available on the adverse impacts of salt stress on pearl millet. In addition, salt tolerance and detoxification mechanisms regulated by different key genes, as reported in other cereals, have not been identified or studied in pearl millet. Pearl millet [*Pennisetum glaucum* (L.) R. Br.] is an important C4 small grain crop grown in the arid and semi-arid regions of Africa and Asia, especially for forage, grain, and stover (Vadez et al., 2012; Khan et al., 2020). It is considered an important cereal crop with excellent nutrient composition (Yadav et al., 2011), as well as a potential biofuel grain feedstock (Lata et al., 2013). Pearl millet is considered as an adaptive crop to various stresses, such as drought, temperature, high pH, and salinity, and its occurrence as an individual or with other crops is of great importance for functional genomics to investigate the deep molecular mechanisms of stress tolerance (Lata et al., 2013; Rajaram et al., 2013). To cope with salt stress, it is crucial to explore the key components of the salt tolerance network in plants (Zhu, 2016), providing a clear understanding of stress signaling and responses. This will upsurge the capability to enhance stress resistance in crop plants to achieve agricultural sustainability and food security.

RNA sequencing is an important and evolving technique that provides an individual’s transcriptome profiling. It makes for easier utilization of next-generation sequencing or deep sequencing for transcriptome analysis (Sudhagar et al., 2018). Transcriptome analysis is a fundamental tool to better understand the underlying molecular pathways controlling the cell in a host (Mutz et al., 2013). Moreover, it is an effective and fast approach to survey the genome, functional identification of genes, transcription factors, and molecular markers (Morozova et al., 2009). In 2017, the genome of pearl millet was reported by Varshney et al. (2017), but lacked complete annotation (Sun et al., 2020). Illumina sequencing is an efficient technique used to quantify gene expression and high-quality reads. However, computational assembly for Illumina technology is required due to the production of short-length reads (Rhoads and Au, 2015; Goodwin et al., 2016). Based on these problems, a single sequencing technology may produce unsatisfactory results. Therefore, we combined both sequencing techniques to identify and explore molecular responses of pearl millet behind salt stress tolerance. Firstly, the raw data of the full-length transcriptome were corrected by using the Illumina sequencing technique. Secondly, the updated full-length transcriptome data were used as a reference to analyze short sequencing data.

Moreover, the molecular mechanisms underlying salt stress tolerance in pearl millet remain unclear. Previous reports show the morphological, physiological, and biochemical changes in pearl millet (Khan et al., 2020), but there are no reports on transcriptomic studies particularly associated with salt stress tolerance through molecular mechanisms. Therefore, to comprehend the molecular mechanisms of salt tolerance in pearl millet, the present constructed the transcriptome profiling of control and salt-treated leaves. In addition, the Gene Ontology (GO), and the Kyoto Encyclopedia of Genes and Genomes (KEGG) analyses were performed to elucidate the important pathways, transcripts, and TFs that could be important to improve tolerance in pearl millet as well as in other cereal crops. The real-time quantitative reverse transcription polymerase chain reaction (qRT-PCR) analysis of important transcripts enriched in important pathways was carried out to validate the transcriptome data.

## Materials and Methods

### Experimental Materials and Growth Conditions

The seeds of pearl millet [*Pennisetum glaucum* (L.) R. Br.] variety “Tifleaf 3” was provided by the College of Grassland Science and Technology, Sichuan Agricultural University, Chengdu, China. The seeds were surface-sterilized with sodium hypochlorite (1%v/v) for 3 min and subjected to wash five times with distilled water (Awan et al., 2020). After that, seeds were air-dried and grown in plastic pots with quartz sand and placed in a growth chamber. These pots were exposed to day and night periods for 14 and 10 h under a temperature of 26 and 22°C, respectively. The pots were fertigated with half-strength of Hoagland’s nutrient solution for 13 days in the growth chamber (Sun et al., 2020). After 13 days, the pots were divided into two groups, i.e., Control and NaCl-treated group. The NaCl treatment was given by dissolving NaCl at 100 mM in half-strength of Hoagland’s nutrient solution.

### Sample Collection and RNA Extraction

Leaf samples of control and NaCl-treated plants were collected at 1, 3, and 7 h post-treatment with six replicates in cryogenic vials and immediately stored at −80°C. The extraction of total RNA was carried out using the RNeasy Plant Mini Kit (QIAGEN, Germany) according to the manufacturer’s protocol. The quality of RNA was assessed through RNA gel electrophoresis.

### Preparation of RNA-Seq Library and Sequencing

A NanoDrop spectrophotometer (California, United States) was used to check out the purity of RNA. The RNA concentration was measured by using a Qubit RNA assay kit in a Qubit 2.0 fluorometer system (California, United States). The libraries were constructed by the NEBNext Ultra™ directed RNA library preparation kit for Illumina. Firstly, the NEBNext Poly (A) mRNA Magnetic Isolation Module was used to enrich the mRNA, then fragment buffer was added to break the mRNA into short fragments, and the cDNA strands were synthesized with random hexamer primers. Secondly, the dNTP, DNA polymerase I, and buffer were added as per the protocol to synthesize the second strand of cDNA (TIANGEN, Beijing). Subsequently, the double-stranded cDNA was purified with the AMPure XP beads, the ends were repaired, the tails were added for sequencing, and the size of fragments was screened with AMPure XP beads. Finally, the cDNA library was obtained by PCR enrichment. The HT DNA high sensitivity assay kit (TIANGEN, Beijing) was used for quality control and quantification of the cDNA library. The RNA-Seq was performed using the Illumina HiSeq2500 (Novogene Bio-Technology, Nanjing, China) as per the manufacturer’s protocol to generate reads for each sample. A total of 18 RNA-Seq libraries were constructed in this study and we required a minimum of 500 ng of total RNA for QC and library preparation for Illumina sequencing. The RNA sequencing of three biological replicates per treatment was carried out.

### Data Analysis

The Illumina sequencing quality control of raw reads was assessed by the FastQC (version 0.11.9).^1^ Each paired-end library has an insert size of 150 bp. Later, the Trimmatic (version 0.36) (Bolger et al., 2014) tool was employed as a command line to filter out the adapters and the low-quality reads of raw reads. Having trimmed raw reads, the quality of filtered data was again evaluated by the FastQC (version 0.11.9), and the clean reads were mapped using the SRR11816223 Pac-bio (Sun et al., 2020) sequencing data as the reference, and the transcriptome index file was constructed through the Kallisto index command (Bray et al., 2016). Likewise, the Kallisto software was used to identify the gene expression level of each sample. Finally, the differential expression analysis was performed by the software tximport and DESeq2 (Wang et al., 2009). The transcripts with an adjusted value of *p* ≤ 0.05 were identified as differentially expressed transcripts (DETs).

### Gene Functional Annotation

Gene functional annotation was carried out by using several online tools such as BLASTX for protein-coding sequences alignment, Swiss-Prot protein^2^; KOG,^3^ protein family (Pfam^4^), KEGG,^5^ GO annotation,^6^ etc. The software GOseq R package (Method Gene ontology analysis for RNA-seq: accounting for selection bias) was used for GO enrichment analysis of DETs (*q* value ≤ 0.05). This software is based on Wallenius’ non-central hypergeometric distribution, which can accurately calculate the probability of GO term being enriched by differential genes. Thereafter, the identified GO terms were used as input in the AgriGO (Du et al., 2010; Tian et al., 2017) for the distribution of GO terms as per the identified DETs. The KEGG enrichment results were obtained by KOBAS 3.0 (Xie et al., 2011) analysis (*P*-value ≤ 0.05). Afterward, the generated KEGG IDs were used as an input in the KEGG database to distribute the DETs in different biological pathways, playing an important role in different metabolic processes.

### cDNA Preparation and Quantitative Reverse Transcription Polymerase Chain Reaction Validation of Differentially Expressed Transcripts

The cDNA synthesis was carried out using RNA samples of pearl millet plants at different treatments of salt stress. The sequences of twelve nominated transcripts were retrieved from the transcriptome data, their primers were designed using the AmplifX 1.5.4 software, and the list of primers used in the qRT-PCR is given in Supplementary Table 1. Ubiquitin was used as an internal control in qRT-PCR; the reaction was performed in a 96-well plate on an ABI prism 7500 Real-Time PCR System using the SYBR Green Master ROX (TaKaRa). The relative expression level of the selected DETs was calculated by following the 2^–ΔΔCT^ method (Livak and Schmittgen, 2001). The reaction was carried out using three biological replicates with three technical replicates.

### Measurement of Oxidative Damage and Antioxidant Enzyme Activity

Leaf sampling of pearl millet seedlings for the measurement of MDA and H_2_O_2_ was carried out at 1, 3, and 7 h of salt treatment. The MDA contents were assessed according to the protocol of Heath and Packer (1968) as lipid peroxidation. In total, 500 mg of fresh leaf tissue samples in triplicates per treatment were taken and homogenized by adding TCA (10%) and 2-thiobarbituric acid (0.65%), followed by heating at 95°C for 60 min. After that, the mixture was kept at room temperature and allowed to cool and then centrifuged at 10,000x ***g*** for 10 min at room temperature. The supernatant was collected, and the absorbance was recorded at 532 nm. The difference in blank and sample absorbance was used to calculate MDA contents. On the other hand, the H_2_O_2_ was determined by using methods described by Khan et al. (2021a). In total, 500 mg fresh leaf tissue samples in triplicates per treatment were taken and homogenized in an ice bath with 5 ml of0.1% (w/v) trichloracetic acid (TCA). The homogenized mixture was centrifuged for 15 min at 12,000x ***g*** at room temperature. After that, 0.5 ml of supernatant was taken and mixed with 1 ml of potassium iodide (1M) and0.5 ml phosphate buffer (10 mM) (pH 7). The absorbance of the mixture was measured at 390 nm. The difference in blank and sample absorbance was used to calculate H_2_O_2_ concentration. In addition, the proline content was measured according to the protocol given by (Bates et al., 1973).

Moreover, to estimate the antioxidant enzyme activities, pearl millet leaf samples were crushed with the help of a pestle and mortar. The crushed material was homogenized with0.5 M phosphate buffer (pH 7.8) and was filtered. The filtrate was subjected to centrifugation for 10 min at 12,000 × *g* at 4°C. The supernatant was separated, collected, and used to measure the activities of antioxidant enzymes. The SOD and POD activities were measured by following the method of Zhang (1992). The CAT activity was estimated according to the protocol described by Aebi (1984). The antioxidant activities are expressed in the unit of U mg^–^^1^.

### Statistical Analysis

All the physiological data of the present study were analyzed by the one-way ANOVA using the statistics 8.1 tool version 2.0 (a commercial software package developed by analytical software). It can be found and downloaded from https://statistix.updatestar.com/en. The data are the values of means ± SD of triplicates per treatment. The least significant difference (LSD) test was used for the comparison among different treatments. The significance level was considered at *p* ≤ *0.05.*

## Results

### Illumina Sequencing of Different Samples and Data Analysis

Initially, the leaves of pearl millet treated with salt stress and control samples were used for RNA extraction. The extracted RNA from both control and salt stress were further prepared for cDNA library preparation for Illumina sequencing. After sequencing, the raw reads were ranged from approximately 6 million to 60 million reads in different samples (Supplementary Table 2). After a quality check, the adapters and low-quality reads were eliminated from raw reads. The clean data were ranged from approximately 6 million reads to 59 million reads in different samples under a Q-phred value of 30%. Furthermore, the GC contents were ranged from 54 to 57%. The mapping data depicted that > 90% reads were successfully mapped to the available pearl millet genome; therefore, this high coverage of the reads makes the sequencing data reliable for secondary bioinformatics analysis. In addition, Supplementary Table 3 contains the details of all transcripts and screened transcripts that were induced in pearl millet leaves under salt stress at 1, 3, and 7 h.

### Identification of Differentially Expressed Transcripts and Their Functional Classification Under Salinity Stress

**FIGURE 1:**
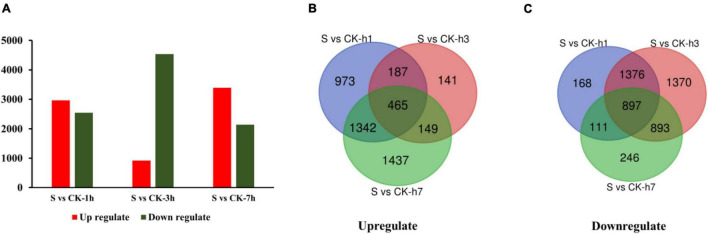
This diagram is showing the expression pattern of DETs among different comparison groups **(A)**. Venn diagram shows the upregulation of DETs among various treated groups **(B)**. Venn diagram shows the downregulation of DETs among different treated groups **(C)**. Here, “S” is indicating salt-treated groups, “CK” is indicating control group, and “h” is used for hours, i.e., 1 h (1 h), 3 h (3 h), and 7 h (7 h).

The comprehensive functional annotation of non-redundant unigenes was obtained by analyzing a total of the 34,086 transcripts against the (Nt) database and the Swiss-Prot database by BLASTN (E-value 1e-10), and among which, 6,141 transcripts were differentially regulated (Supplementary Table 4). Pairwise comparisons such as S vs. CK-1 h, S *vs.* CK-3 h, and S *vs.* CK-7 h showed commonly annotated 1,864 DETs. Among these comparisons, 2,967 transcripts were upregulated, 2,552 transcripts were downregulated at S vs. CK-1 h, 942 upregulated and 4,536 downregulated at S vs. CK-3 h, and 3,393 upregulated, and 2,147 downregulated at S *vs.* CK-7 h treatment (Figure 1 and Supplementary Table 4).

### Gene Ontology Analysis

**FIGURE 2:**
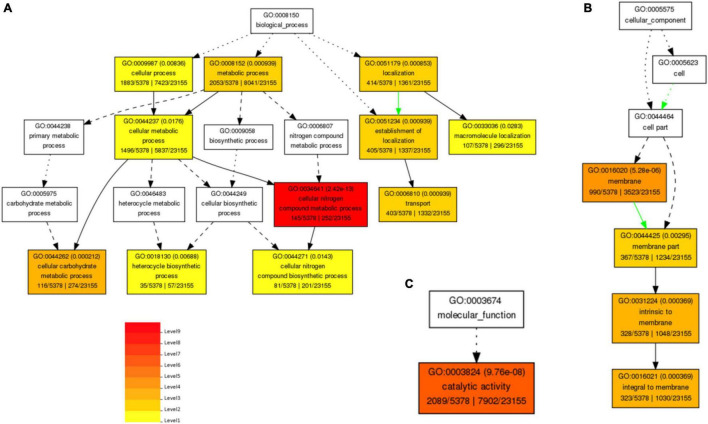
Biological process-based GO enriched terms **(A)**, and Cellular component-based enriched GO terms **(B)** and molecular function-based Enriched GO term **(C)** in pearl millet seedlings under salt stress. Red to yellow colors exhibit the high to low enrichment of DETs in each GO term.

Gene Ontology (GO)-based enrichment analysis revealed that annotated transcripts were classified into three functional categories: biological process, molecular functions, and cellular components, and clustered into 24 classifications (Figures 2A–C). The total number of DETs among different time points in pearl millet under salt stress is annotated in GO analysis as presented in Supplementary Table 5. These DETs were involved in the biological, molecular, and cellular functions. The biological process category contains 17 classifications, and the largest subcategory was “metabolic process” (GO: 0008152), whereas the second largest subcategory was “cellular process” (GO: 0009987). Moreover, in the molecular function category, the only subcategory was “catalytic activity” (GO: 0003824), and in cellular component categories, there were 6 classifications. Among these, the largest subcategory was “membrane” (GO: 0016020), and the remaining subcategories were “membrane part” (GO: 0044425), intrinsic to “membrane” (GO: 0031224), and “integral to membrane” (GO: 0016021).

### Kyoto Encyclopedia of Genes and Genomes Analysis

**FIGURE 3:**
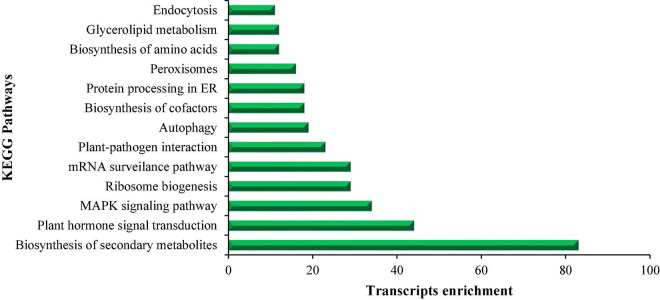
This figure shows the list of KEGG pathways enriched in salt treatment at different time points (S *vs.* Ck 1 h, 3 h, and 7 h) and transcript enrichment in these pathways.

The sequencing data of DETs were used as input in KOBAS (Xie et al., 2011) (*P*-value ≤ 0.05) for retrieving KEGG IDs, and these KEGG IDs were employed for constructing the pathways. The KEGG pathway analysis revealed the enrichment of DETs in different biological pathways at different time periods. The commonly enriched pathways are metabolic pathways, biosynthesis of secondary metabolites, plant hormone signal transduction, mitogen-activated protein kinase (MAPK) signaling pathway, ribosome biogenesis, mRNA surveillance pathway, plant-pathogen interaction, autophagy, biosynthesis of cofactors, protein processing in ER, peroxisomes, biosynthesis of amino acids, glycerolipid metabolism, and endocytosis as shown in Figure 3. The KEGG pathways were achieved based on a value of *p* ≤ 0.05. However, the most significant pathways under salt stress are the “MAPK signaling pathway” and “peroxisome pathway” as they were enriched among all treatments.

### Mitogen-Activated Protein Kinase Signaling Pathway

**FIGURE 4:**
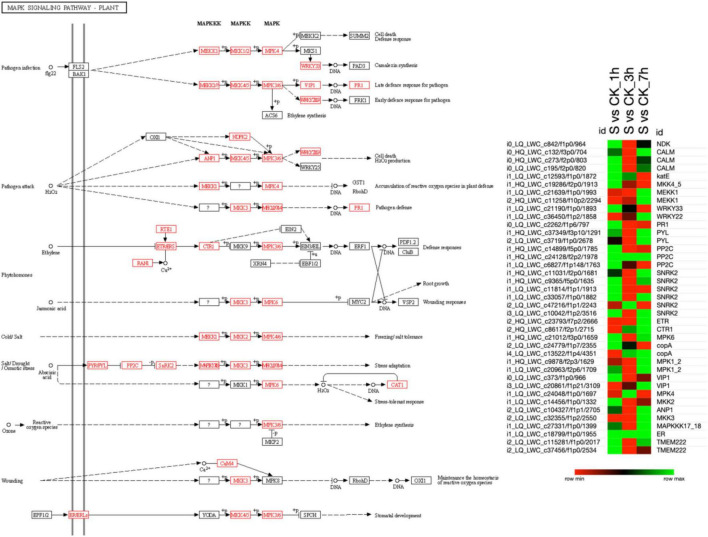
The MAPK signaling pathway shows the differences in the expression patterns among different combinations of salt treatment at different time points. All the identified DETs involved in that pathway are shown in red color and heatmap illustrating the expression pattern of transcripts at 1, 3, and 7 h of salt treatment. Here, the green color in the heatmap shows the upregulation of DETs, and the red color shows the downregulation when the DETs were compared among various combinations, as mentioned on the top of each column. Moreover, “S” indicates salt-treated groups, “CK” indicates the control group, and “h” is used for hours, i.e., 1 h (1 h), 3 h (3 h), and 7 h (7 h).

The KEGG analysis revealed that the MAPK signaling pathway was the most enriched pathway in which several DETs participated in various stress responses, i.e., pathogen infection, pathogen attack, phytohormones, cold/salt stress, salt/drought/osmotic stress, ozone, and wounding (Figure 4). The results revealed that the KEGG database screened out 39 DETs participating in the MAPK pathway (Supplementary Table 6). A higher proportion of DETs at 1 and 7 h of salt treatment was found to be upregulated. However, at 3 h of salt treatment, their expression was downregulated, which may lead to the prompt signaling response of pearl millet on exposure to salt stress compared to control plants.

The heat map in that figure shows the upregulation and downregulation of different DETs involved in the MAPK pathway under salt at different time points. There were 26 DETs upregulated at 1 h of salt *vs.* ck, 7 DETs upregulated at 3 h of salt *vs*. ck, and 24 DETs were upregulated at 7 h of salt *vs.* ck. However, only two DETs (i1_HQ_LWC_c24128/f2p2/1978) and (i1_HQ_LWC_c18799/f1p0/1955) were commonly upregulated among these three combinations. In addition, the remaining DETs in this heat map were found downregulated, as shown in Figure 4.

Overall, most of the DETs involved in the pathogen infection, pathogen attack, and phytohormones, except ethylene, cold/salt stress, and salt/drought/osmotic stress, were upregulated and downregulated wounding and ethylene-based responses to salt stress. Therefore, it can be concluded that early and late exposure to salt stress activates a variety of DETs in plants that actively participate in stress responses. In addition, most of the DETs are involved in abiotic stress responses that might play an important role in enhancing the plant resistance against abiotic stresses.

### Peroxisome Pathway

**FIGURE 5:**
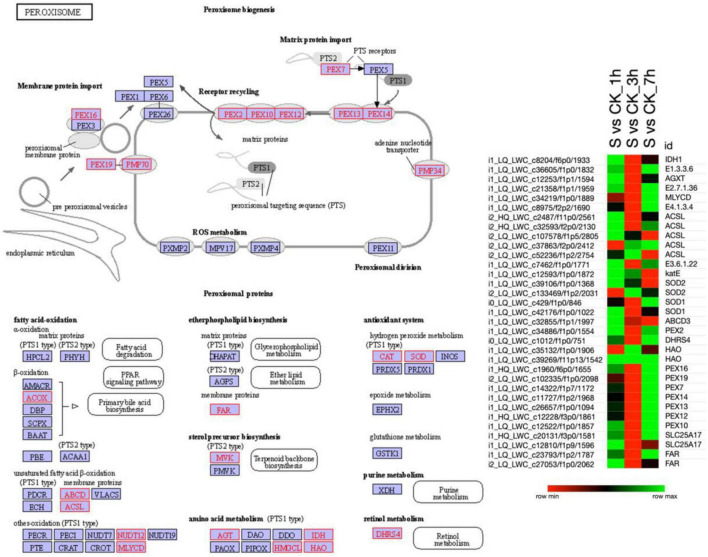
The KEGG analysis shows enrichment of DETs involved in the “Peroxisomal pathway”. Red color boxes are exhibiting the involvement of DETs in the peroxisome pathway, whereas, the heatmap illustrates the expression pattern of DETs in pearl millet leaves under salt stress. Here, the green color in the heatmap shows the upregulation of DETs and the red color shows the downregulation when the DETs were compared among various combinations as mentioned on the top of each column. Moreover, “S” is indicating salt-treated groups, “CK” is indicating the control group, and “h” is used for hours, i.e., 1 hour (1 h), 3 hours (3 h), and 7 hours (7 h).

Similar to the MAPK signaling pathway, the KEGG database revealed the 33 DETs that were strongly involved in the peroxisome pathway (Figure 5 and Supplementary Table 7). According to the results, isocitrate dehydrogenase (IDH1), acyl-CoA oxidase (E1), alanine-glyoxylate transaminase (AGXT), mevalonate kinase (E2), etc., were upregulated at 1 h of salt treatment. Besides, at 7 h of salt treatment, most of the S-2-hydroxy-acid oxidase (HAO) and peroxin (PEX) were upregulated, and the rest of the transcripts seemed to be downregulated. Furthermore, as compared to 1 and 7 h of salt treatment, a higher proportion of DETs were downregulated in 3 h of salt treatment. Overall, the results indicated that most of the DETs in the peroxisome pathway were upregulated and a few downregulated in 1 and 7 h and vice versa in 3 h of salt treatment suggesting the defensive response of pearl millet under immediate exposure to salt stress.

### Differentially Expressed Transcription Factors

Plant transcription factors (TFs) are highly responsible for regulating the various physiological programs crucial for plant survival. In the present study, 140 TFs belonging to different TFs families, such as (AP2-ERF; 7), (bHLH; 17), (ERF; 7), (HBP; 10), (HSF; 5), (MADS; 14), (MYB; 11), (NAC; 4), (WRKY; 15), etc., were differentially regulated on exposure to salt treatment (Figure 5 and Supplementary Table 8). Most of the TFs were found to be upregulated at 1 and 7 h of salt treatment and further revealed the downregulation at 3 h of treatment. Among the seven AP2-ERF TFs family members, 5 TFs were upregulated in 1 h, 6 in 7 h, and 1 in 3 h of treatment, whereas, the 6 TFs in 3 h treatment were highly downregulated among the total of seven TFs. Only one AP2-ERF TF (XP_004965682.1) was upregulated in all treatments. Similarly, at 3 and 7 h of salt treatment, most of the TFs were upregulated, and less in number were downregulated. By comparing all salt treatments, a large number of bHLH, ERF, HBP, HSF, MADS, MYB, NAC, and WRKY TFs were significantly upregulated at 7 h treatment than 1 and 3 h of salt treatment.

Among these TFs, 13 bHLH, 4 ERF, 9HBP, 10 MAD, 9 MYB, 4 NAC, and 8 bHLH were highly upregulated at 7 h of salt treatment as compared to 1 and 3 h of salt treatment. Overall, several DETs were significantly upregulated and downregulated and have specified different functions in pearl millet under salt stress. As 18 TFs were referred to thioredoxin, 1 SA, 195 receptor kinases, 307 protein modification, 372 protein degradation, 18 phosphoinosetoids, 4 periredoxin, 16 MAPK, 21 light, 11 JA, 41 IAA, 3 heme, 5 glutaredoxin, 6 GA, 63 G-protein, 24 ethylene, 6 dismutases, 3 cytokinins, 60 calcium regulation, 6 C and nutrition, 11 BA, 20 ascorbate glutathione, 19 ABA, and 5 TFs are unspecified (functions not identified).

### Plant Hormones

**FIGURE 6:**
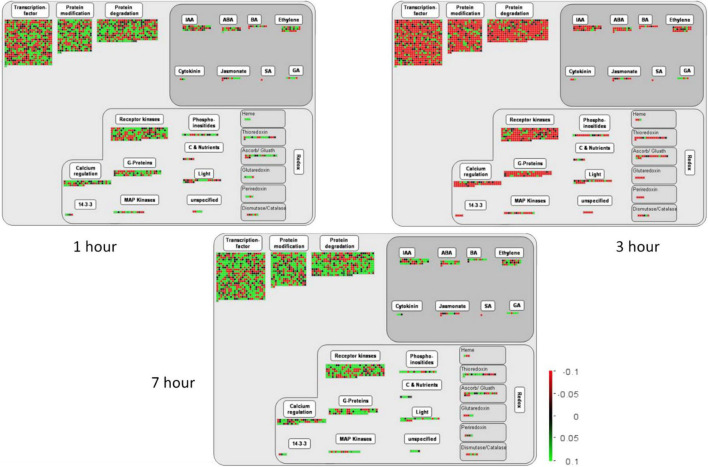
This figure exhibits the MapMan expression analysis of various DETs involved in several molecular and cellular functions at 1, 3, and 7 h of salt treatment in pearl millet. Here, the green color in the heatmap shows the upregulation of TFs and DETs, and the red color shows the downregulation when the TFs and DETs were compared among various combinations as mentioned on the top of each column. Moreover, “S” indicates salt-treated groups, “CK” indicates the control group, and “h” is used for hours, i.e., 1 hour (1 h), 3 hours (3 h), and 7 hours (7 h).

Plant hormones significantly influence plant morphology and physiology (Kong et al., 2020; Khan et al., 2021a). They are majorly involved in growth, development, signaling, and resistance against biotic and abiotic stresses (Nawaz et al., 2020). The transcriptome data of the present study reveal the important TFs related to biosynthesis and the regulation of different hormones such as salicylic acid (SA), jasmonic acid (JA), indole acetic acid (IAA), gibberellic acid (GA), ethylene (ET), brassinosteroids (BRs), and abscisic acid (ABA) (Figure 6).

Auxins play a crucial role in various growth and behavioral processes during the plant life cycle and are critically involved in organ development in plants (Uggla et al., 1996). The present study detected 41 auxins responsive TFs induced after 1, 3, and 7 h of salt treatment. However, most are upregulated at 7 h and downregulated at 1 and 3 h of salt treatments. In addition, ABA is also involved in regulating various metabolic functions, especially signal perception, signal transduction, and stress response and known as stress hormone (Nawaz et al., 2020). Moreover, about 19 ABA differentially expressed TFs in pearl millet seedlings were under salt stress. At 3 h of salt treatment, the expression of ABA TFs was found to be downregulated followed by 1 and 7 h. The upregulation of ABA-responsive TFs at 7 h may indicate the severity of salt stress in later stages. According to previous reports, ET also participates in the physiological mechanism as well as stress resistance in plants (Kong et al., 2020). The present study identified 24 differentially expressed ethylene TFs and seemed to be downregulated at 3 h of salt treatment. The results showed that 1 and 7 h of salt treatment highly upregulated the ET-related TFs, which might be involved in signaling pathways. Besides, auxins, ABA, and ET hormones, 11 JA, 6 GA, and 11 BA responsive TFs were also identified in pearl millet under salt stress. Previous literature reported that these hormones are actively involved in the growth and yield of plants (Kong et al., 2020). Interestingly, most of these TFs were upregulated at 1 and 7 h and downregulated at 3 h of salt treatment, as found in Figure 6. The transcriptional regulation in pearl millet at 1, 3, and 7 h of salt treatment were provided in Supplementary Table 9.

### Kinases

Protein kinases perform a crucial role in the growth and development of plants and are strongly involved in tolerance to various environmental stresses (Cui et al., 2018). Receptor-like kinases (RLKs) are the important class of protein kinases that actively participate in perceiving the external signals and stimulating intercellular reactions under stress conditions. The present study identified 195 receptor kinases and 16 MAPKs in pearl millet under salt stress at different time points. Interestingly, the results of the present study indicated that salt stress at 1 and 7 h upregulated most of the RLKs and MAPKs whereas; their expression was downregulated at 3 h of salt treatment (Figure 6 and Supplementary Table 10). The upregulation of these RLKs and MAPKs might reflect their important role in salt stress. In addition, the details about DETs that were involved in protein modification, protein degradation, hormonal regulation, kinases, and receptor kinases through m analysis are given in Supplementary Table 10.

### RNA-Seq Data Validation

Eight randomly selected DETs were evaluated for their expression patterns at different time points to validate the RNA-Seq data (Supplementary Figure 1). The sequences of transcripts were retrieved from the transcriptome data. The transcript i1_LQ_LWC_c22131/f1p5/1616 was highly upregulated at 7 h of salt treatment as compared to other transcripts. Furthermore, the trend of upregulation was found more at 1 and 7 h of salt treatments and less at 3 h of salt treatment than control. The expression profile of DETs was followed as 1 h, 7 h, and then 3 h of salt treatment. In short, the qRT-PCR results validated the expression pattern of selected DETs mentioned in RNA-Seq data.

### Oxidative Damage and Antioxidant Enzyme Activities

**FIGURE 7:**
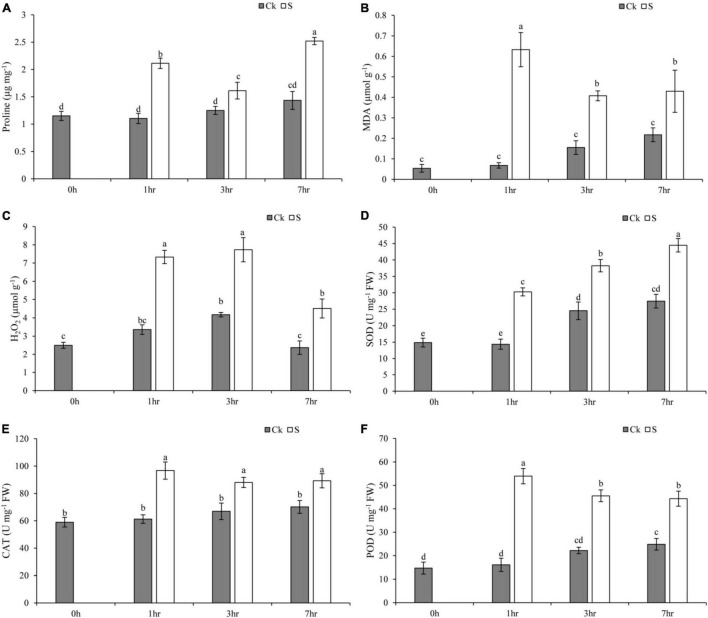
This figure shows the impact of salt stress on **(A)** proline content, **(B)** MDA content, **(C)** hydrogen peroxide (H_2_O_2_), **(D)** SOD, **(E)** CAT, and **(F)** POD at 1, 3, and 7 h for control (Ck) and salt-treated seedlings (S). Values are the mean of three replicates and represented as mean ± SD of three biological replicates.

Salt stress negatively impacted plant morphological and physiological profile as compared to the control group. Moreover, the morphological presentation of pearl millet seedlings after 7 days of salt treatment clearly showed the damaging impact of salt stress on leaves (Supplementary Figure 2). In addition, the physiological profile of the present study depicted that salt stress at 1, 3, and 7 h of treatment increased the oxidative damage in terms of MDA and H_2_O_2_. However, the activities of antioxidant enzymes were also regulated in pearl millet seedlings (Figure 7). The pearl millet seedlings exhibited a significant increase in proline content at 1 and 7 h of salt treatment, whereas control (Ck) seedlings showed a gradual and smooth trend concerning an increase in proline as compared to a 0-hr treatment (Figure 7A). In addition, MDA content and H_2_O_2_ concentration were also enhanced at 1, 3, and 7 h of salt treatment which may be due to sudden exposure of salt to pearl millet seedlings (Figures 7B,C). The H_2_O_2_ concentration seemed to decrease at 7 h of salt treatment as compared to 1 and 3 h. The control (Ck) seedlings at 1, 3, and 7 h showed an increased level of MDA and H_2_O_2_ when compared with 0-h seedlings. Moreover, salt treatment enhanced the activities of SOD, CAT, and POD at all time points (Figures 7D–F). The maximum enhancement of SOD activity was recorded at 7 h, while CAT and POD activities were recorded at 1 h of salt treatment as compared to other time points. However, the control seedlings at all time points showed a gradual increase in the activities of SOD, CAT, and POD. The raw data of physiological parameters of pearl millet seedlings under salt stress at 1, 3, and 7 h of treatment were given in Supplementary Table 11.

## Discussion

The development and utilization of high-throughput sequencing technologies efficiently uncover the molecular basis of stress response and assist in the understanding of genome-wide expression patterns of genes in sorghum under abiotic stress (Zhang et al., 2010; Gelli et al., 2014). The RNA-Seq technology is an effective tool to discover genes and their associated pathways involved in salinity responses in plants through expression profiling. In the present study, transcriptome profiling of a pearl genotype was performed under salt stress at three different time points, i.e., 1, 3, and 7 h after salt treatment.

### Regulation of Differentially Expressed Transcription Factors

**FIGURE 8:**
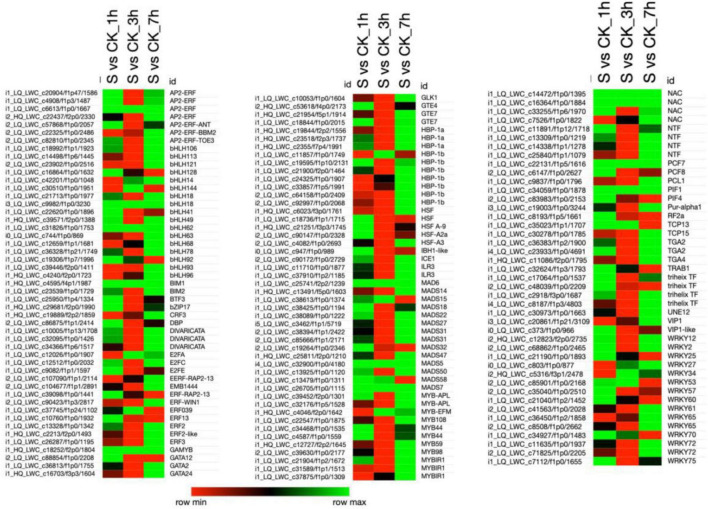
This figure shows the heatmap that represents the differentially expressed transcription factors (TFs) that belong to different TF families in pearl millet and are expressed under salt stress. The data of TFs were retrieved from the plant transcription factor database (PTFDB) (http://planttfdb.gao-lab.org/). Here, the green color in the heatmap shows the upregulation of TFs and the red color shows the downregulation when the TFs were compared among various combinations as mentioned on the top of each column. Moreover, “S” indicates salt-treated groups, “CK” indicates the control group, and “h” is used for hours, i.e., 1 hour (1 h), 3 hours (3 h), and 7 hours (7 h).

In the present study, different DET-encoding TFs were identified, which were upregulated or downregulated under salinity stress. Most of the TFs were upregulated at 1 and 7 h of salt treatment, which might have an effective role in signaling pathways, metabolic mechanisms, and developmental processes in pearl millet (Figure 8). For example, bHLH18, bHLH49, and bHLH62 were highly upregulated at 1 and 7 h of salt treatment. *CsbHLH18* is a key player in cold stress that minimizes the level of ROS to balance the cell homeostasis and promote growth in *Citrus sinensis* (Geng and Liu, 2018). *TabHLH49* positively enhances the expression level of the dehydrin *WZY2* gene under drought and improves wheat growth (*Triticum aestivum* L.) plants (Liu et al., 2020). *CmbHLH62* plays an important regulatory role in photoperiodic responses in (*Cucumis melo* L.) (Ren et al., 2016). Moreover, transcription factor *GBOF-1* is an alternative name of *bHLH62* responsible for drought stress responses in tomatoes (*Solanum lycopersicum*) (Gong et al., 2010). Several WRKY transcription factors were also detected from the RNA-Seq, e.g., WRKY2 and WRKY12 were highly upregulated at 1 and 7 h of salt treatment. Similarly, *HvWRKY2* involves in the defense mechanism of plants and also enhances the resistance to pathogenic infection in barley (*Hordeum vulgare*) (Mangelsen et al., 2008). *GmWRKY12* actively participates in the growth and development of soybean (*Glycine max*) plants under salt and drought stress by minimizing the level of MDA and ROS (Shi et al., 2018). In addition, MYB TFs such as *MYB59*, *MYB98*, and *MYB108* were seemed to be highly upregulated at 7 h of salt treatment. Likewise, *MYB59* represses and activates the different defensive pathways in plants, involved in the heat, drought, and salinity stress tolerance in Arabidopsis (Fasani et al., 2019). Previously, it has been described that *MYB98* is upregulated on exposure to oxidative stress and regulates the physiological mechanisms of plants (Han et al., 2014). Existing studies indicate that *MYB108* seems to be upregulated in Arabidopsis roots by salinity and is also found to be involved in abiotic and biotic stress responses (Xu et al., 2019). Furthermore, several MAD-box TFs such as MAD6, MADS5, and MADS7, upregulated among all time points under salt stress. Some evidence exhibit that MADS7 regulates the downstream target genes responsible for anther and pollen development, which ultimately control crop fertility (Jin et al., 2020). Similarly, many ERF TFs that play a significant role in plant growth and development in response to ABA signaling have been identified (Kong et al., 2020). From our study, it can be suggested that salt stress at different time points significantly influenced the transcriptome profile of pearl millet resulting in the upregulation of many TFs responsible for salt resistance in plants. This study provides new insights for researchers to elaborate on deep functional mechanisms of newly identified TFs under various environmental stresses. Moreover, different developmental pathways will show their actual role in the regulation of linkage genes.

### Regulation of Plant Hormones

Plant hormones play a crucial role in the growth and development of plants. Moreover, they are also responsible for plant resistance to various abiotic and biotic stresses (Reinhardt et al., 2000). The present study identified important TFs related to the biosynthesis of IAA, ABA, BA, JA, SA, GA cytokinin, and ET (Figure 6). Most of the plant hormone-related TFs were upregulated at 1 and 7 h of salt treatment. However, they are downregulated at 3 h, as shown in the active participation of plant hormones-related TFs in hormonal signaling mechanisms. Auxins, a class of plant hormones, are strongly involved in the developmental and behavioral processes in plants and are important for plant’s body growth (Uggla et al., 1996). There were 42 auxin-responsive DETs induced among all time points under salt stress and were highly upregulated at 7 h of salt treatment. In addition, ABA is usually involved in the diverse regulatory functions that control its biosynthesis, degradation, and signal transduction (Xiong et al., 2012). A total of 21 ABA-responsive TFs from the transcriptome data were highly upregulated at 1 and 7 h and downregulated at 3 h of salt treatment. Previous literature showed that cytokinins and GA enhanced the growth and yield of plants (Werner et al., 2001; Wang et al., 2014). In the present study, 3 cytokinins and 6 GA TFs were significantly induced under salt stress at all-time points. Besides auxins, ABA, GA, and cytokinins, the present study identified 28 ethylene and 11 brassinosteroids (BA)-responsive TFs. Similarly, these are highly upregulated at 1 and 7 h and downregulated at 3 h of salt treatment. Therefore, it can be concluded that overexpression or underexpression of plant hormone-responsive DETs might have a direct or indirect role in developmental processes, leading to better plant growth under stress conditions.

### Involvement of Redox Pathway Under Salt Stress

Besides, the results of the current study disclosed the strong expression of DETs involved in plant redox states (Figure 6). Salinity stress leads to severe oxidative stress, and plants need to maintain water status, ion homeostasis, and redox homeostasis to acclimatize salt stress (Hossain and Dietz, 2016). Previously many genes have been identified and characterized in plants that are potentially involved in signal transduction, membrane transport, and redox reactions (Zhang et al., 2008). The Redox system of plants consists of a ROS generation system, an antioxidant defense system, and a redox regulatory network. Similar to our experiment, the expression of different gene families induced by TFs and these genes have encoded CAT, SOD, POX, the ASC-GSH cycle enzymes [APX, monodehydroascorbate reductases (MDHAR), dehydroascorbate reductases (DHAR), glutathione reductases (GR), glutathione peroxidases (GPX), peroxiredoxins (PRX), and glutathione S-transferases (GST) (Mittler et al., 2004; Munns and Tester, 2008; Azevedo Neto et al., 2010)]. In addition, Thiol peroxidases use thiol electron donors, such as thioredoxin (TRX), glutaredoxin (GRX), glutathione, or in rare cases ascorbate, to convert H_2_O_2_ to H_2_O. APX catalyzes the primary step in the classical water-water cycle (Asada, 1992). In the present study, most of the TFs were differentially expressed, showing their participation in reducing oxidative damage in plants. For instance, WRKY25 acts as a redox-sensitive upstream regulatory factor of WRKY53 expression. Under non-oxidizing conditions, WRKY25 binds to a specific W-box in the WRKY53 promoter and acts as a positive regulator of *WRKY53* expression in a transient expression system using Arabidopsis protoplasts, whereas oxidizing conditions dampened the action of WRKY25 (Doll et al., 2020).

### Receptor Kinases

In the present study, a huge number of receptor kinases were highly upregulated on exposure to salinity stress (Figure 6). Generally, the receptor kinases contribute to abiotic stress tolerance in plants (Jose et al., 2020). For instance, *FERONIA* (FER), a member of the *CrRLK1L* family, plays a crucial role in ABA and salt stress responses. Moreover, it has been reported that different receptor kinases, i.e., FER, LRXs, and RALFs, form a signaling network that regulates plant growth by conferring tolerance to salt stress (Zhao et al., 2018). Similarly, salt stress also influenced the expression of MAPK genes to regulate the signaling pathways in response to stress conditions. A MAPK cascade comprises MAP kinase kinases (MAP3Ks/MAPKKKs/MEKKs). In stress conditions, the stimulated plasma membrane activates MAP3Ks or MAP kinase kinase kinase kinases (MAP4Ks). The MAP3Ks are serine/threonine kinases able to phosphorylate a wide range of substrates, including other kinases and transcription factors that enhance plant tolerance to abiotic stress (Sinha et al., 2011) that is similar to our results.

Overall, the RNA-Seq data identified many DET-encoding genes involved in different biological functions under salt stress at different time points. The expression of most of the DETs was upregulated on exposure to 1 and 7 h but downregulated at 3 h. The results of the present study concluded that salinity stress boosted the expression of DETs that have a vast role in salt stress tolerance in pearl millet and need to be explored.

Salt stress negatively impacted the plant growth, physiological, and biochemical profiles. The MDA as lipid peroxidation is considered an important indicator of oxidative damage caused by salinity (Kumar et al., 2021). Sudden exposure to salt stress leads to immediate enhancement of MDA and ROS that reflects the internal imbalance of cells in plants especially the salt-sensitive species (Sarker and Oba, 2020). Sodium and chloride ions lead to significant damage of cell membrane and disturbance of internal homeostasis. In that case, accumulation of proline is found beneficial for plants to maintain internal osmotic balance and provide protection against severe mechanical injury under MDA and ROS production (Rahneshan et al., 2018; Alzahrani et al., 2019). Therefore, the results of the present study depicted an increased level of proline under salinity stress, which might have regulated the impact of MDA and H_2_O_2_ in pearl millet seedlings. In addition, the improved activities of antioxidants have been reported to protect plants against serious effects of MDA and ROS and enhanced plant tolerance to salt stress (Kumar et al., 2021). The results of the present study showed a significant enhancement of SOD, CAT, and POD under salt stress at different time points (1, 3, and 7 h). Antioxidants are the first line of defense in plants against salinity and other stresses. Previously, various plant species showed an increased level of SOD, CAT, and POD under salinity stress to improve plant tolerance to stress (Ali et al., 2017; Sahin et al., 2018; Sarker and Oba, 2020). Hence, the physiological and transcriptomic study revealed that pearl millet plants can withstand salt stress by the regulation of DETs and improvement of antioxidant enzyme activities at the seedling stage.

## Conclusion

In conclusion, the transcriptomic data of the present study identified and interpreted a large number of DETs involved in salt stress in pearl millet, which provides new insights for researchers to carry out genetic and functional analysis in future research work. Different TFs related to salt tolerance, and their expression pattern at 1, 3, and 7 h, distinguished their potential role on early and late exposures to salt stress. In addition, pearl millet seedlings regulate antioxidant enzyme activities to control oxidative damage in terms of MDA and H_2_O_2_, which might be due to the activation or regulation of different TFs and DETs upon salt stress. This study will provide molecular mechanisms behind the salt tolerance of pearl millet and other cereals. Furthermore, molecular breeding of candidate genes from current results will help to improve salt resistance in cereal crops. The highly upregulated and downregulated DETs, their functional annotation, and various signal transduction pathways by KEGG suggested their active contribution to salt tolerance. Overall, the present study results can be used as a reference to identify the novel TFs that play a crucial role in enhancing salt tolerance in crop plants.

## Data Availability Statement

The original contributions presented in the study are publicly available. This data can be found here: National Center for Biotechnology Information (NCBI) BioProject database under accession number SRR11816223.

## Author Contributions

SA and LH designed this project. SA and IK participated in experimental processing and collecting material and wrote the original manuscript. LH supervised the project. SA and RT analyzed the data. MR, LH, XW, and XZ contributed to reviewing and editing the manuscript. All authors contributed to the article and approved the submitted version.

## Conflict of Interest

The authors declare that the research was conducted in the absence of any commercial or financial relationships that could be construed as a potential conflict of interest.

## Publisher’s Note

All claims expressed in this article are solely those of the authors and do not necessarily represent those of their affiliated organizations, or those of the publisher, the editors and the reviewers. Any product that may be evaluated in this article, or claim that may be made by its manufacturer, is not guaranteed or endorsed by the publisher.

## Footnotes

1
http://www.bioinformatics.babraham.ac.uk/projects/fastqc/

2
http://www.expasy.org/sprot/

3
http://www.ncbi.nlm.nih.gov/COG/

4
http://pfam.sanger.ac.uk/

5
http://www.genome.jp/kegg

6
http://www.geneontology.org
