# Supplementary material for: Genome-Wide Expression and Physiological Profiling of Pearl Millet Genotype Reveal the Biological Pathways and Various Gene Clusters Underlying Salt Resistance
Source: Front Plant Sci. 2022 Mar 28;13:849618. doi: 10.3389/fpls.2022.849618 (PMC8996197; doi:10.3389/fpls.2022.849618)
Supplement: Supplementary file 12 [file Data_Sheet_1.docx]

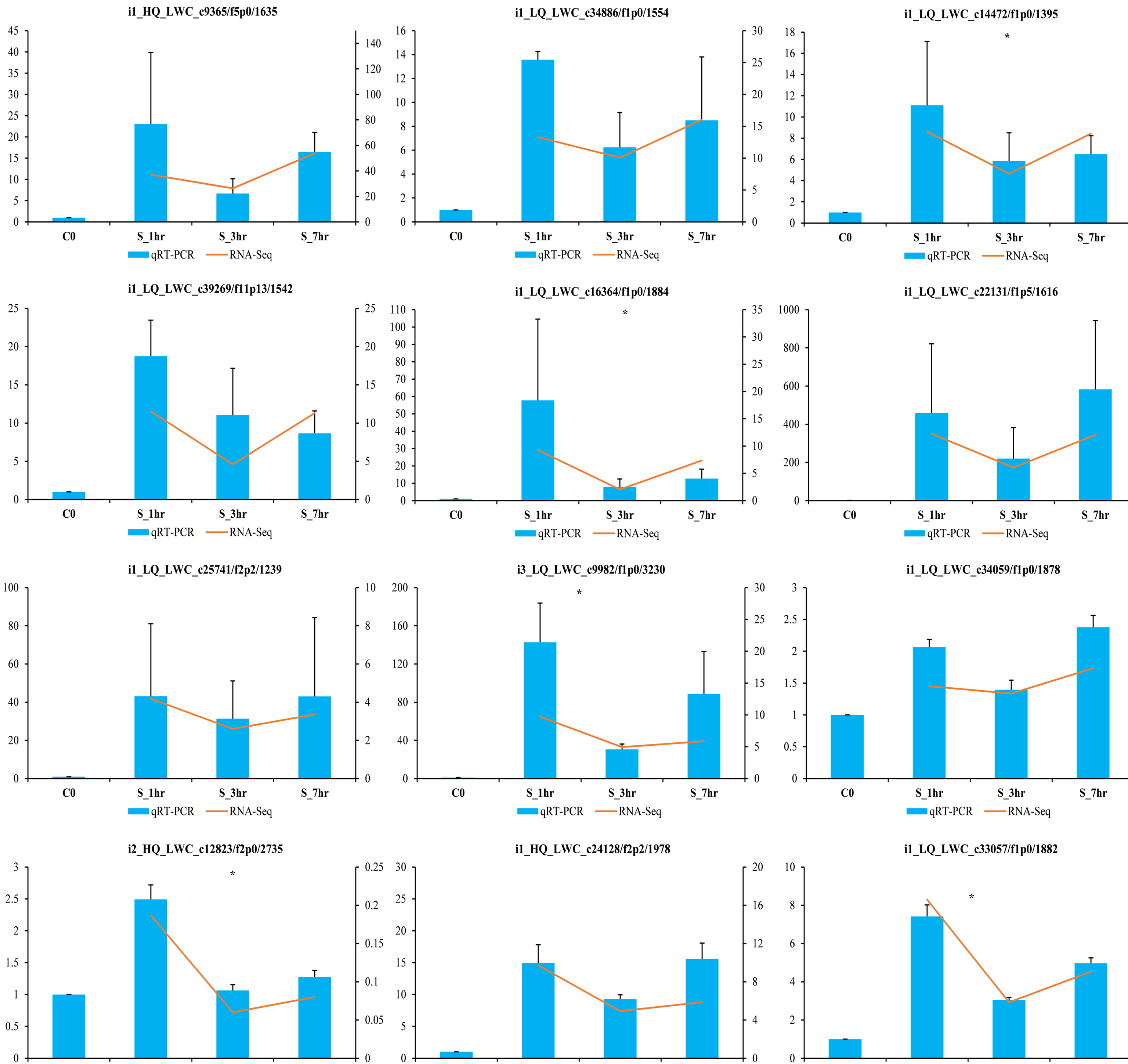


**Supplementary Figure 1.** qRT-PCR based expression pattern of the eight randomly selected DETs from RNA-Seq data. Ubiquitin was used as an internal control in reaction; data are represented as mean ± SD of three biological replicates. The star (*) on bars is indicating the significant differences between or among different treatments under salt stress.
