# Supplementary material for: Genome-Wide Expression and Physiological Profiling of Pearl Millet Genotype Reveal the Biological Pathways and Various Gene Clusters Underlying Salt Resistance
Source: Front Plant Sci. 2022 Mar 28;13:849618. doi: 10.3389/fpls.2022.849618 (PMC8996197; doi:10.3389/fpls.2022.849618)
Supplement: Supplementary file 13 [file Data_Sheet_2.docx]

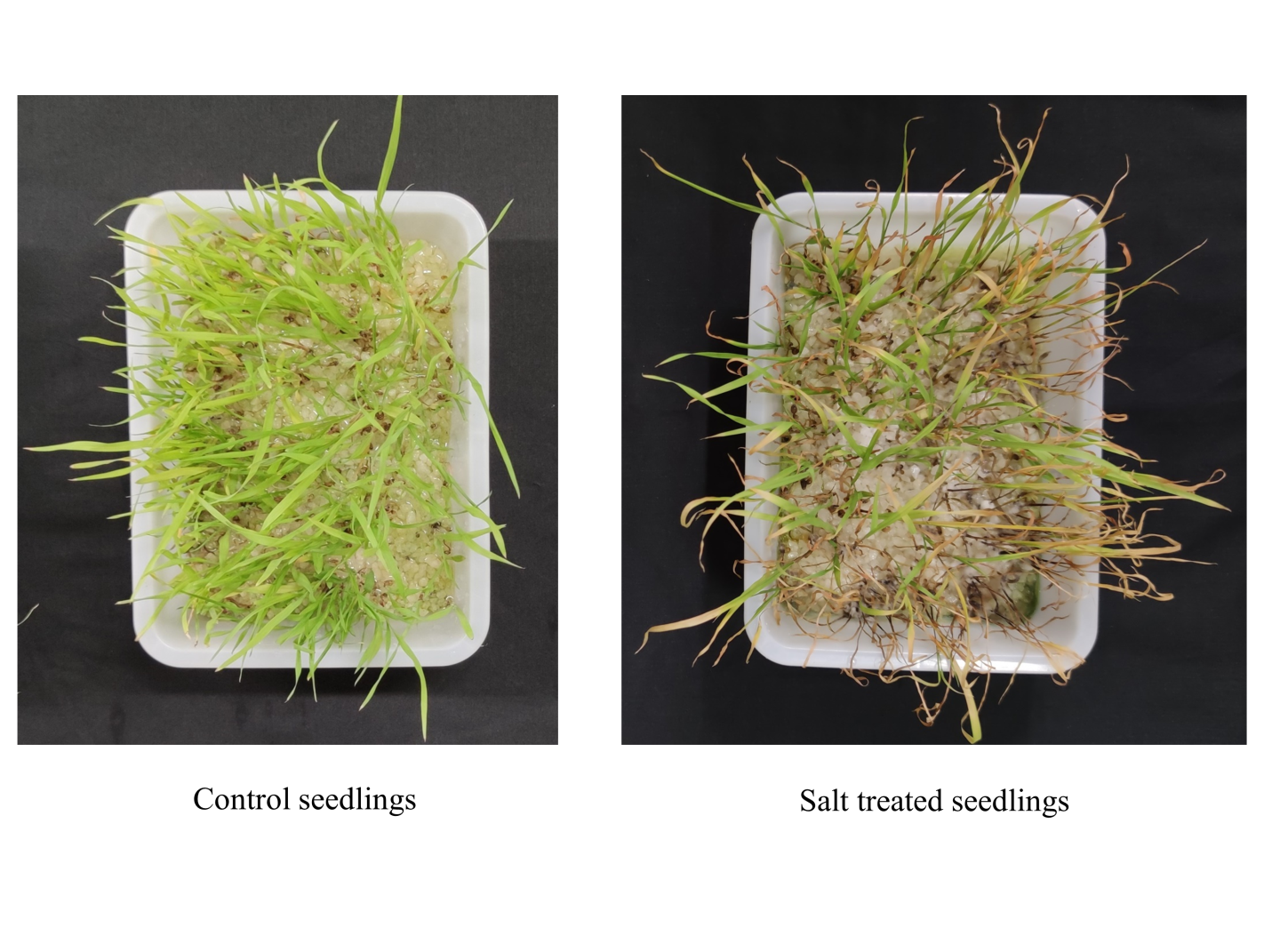


**Supplementary Figure 2.** This figure shows the morphological differences between control and salt treated pearl millet seedlings after seven days of salt treatment.
